# Competitive biosorption of lead, cadmium, copper, and arsenic ions using algae

**DOI:** 10.1007/s11356-012-1208-2

**Published:** 2012-10-02

**Authors:** Abbas H. Sulaymon, Ahmed A. Mohammed, Tariq J. Al-Musawi

**Affiliations:** Environmental Engineering Department, College of Engineering, Baghdad University, Baghdad, Iraq

**Keywords:** Competitive biosorption, Algae, Ion exchange model, Affinity constant, Kinetics

## Abstract

The present study aims to evaluate the competitive biosorption of lead, cadmium, copper, and arsenic ions by using native algae. A series of experiments were carried out in a batch reactor to obtain equilibrium data for adsorption of single, binary, ternary, and quaternary metal solutions. The biosorption of these metals is based on ion exchange mechanism accompanied by the release of light metals such as calcium, magnesium, and sodium. Experimental parameters such as pH, initial metal concentrations, and temperature were studied. The optimum pH found for removal were 5 for Cd^2+^ and As^3+^ and 3 and 4 for Pb^2+^ and Cu^2+^, respectively. Fourier transformation infrared spectroscopy analysis was used to find the effects of functional groups of algae in biosorption process. The results showed that Pb^2+^ made a greater change in the functional groups of algal biomass due to high affinity to this metal. An ion exchange model was found suitable for describing the biosorption process. The affinity constants sequence calculated for single system was *K*_Pb_ > *K*_Cu_ > *K*_Cd_ > *K*_As_; these values reduced in binary, ternary, and quaternary systems. In addition, the experimental data showed that the biosorption of the four metals fitted well the pseudo-second-order kinetics model.

## Introduction

Heavy metal pollution has become a major issue in many countries because their existence in drinking waters and wastewaters often exceed the permissible standards (Ahmet and Mustafa 2008). Metal ions in the environment are bio-magnified in the food chain and are accumulated in tissues; therefore, toxic effects of heavy metals in particular are especially found in animals of higher trophic levels and especially in human. Heavy metals discharged into the aquatic environment will be bound predominantly to suspended materials and finally accumulate in the sediment. The most direct potential routes of human exposure to such discharged metals into a river would be any consumption of water or fish or other food derived from the river.

The metals hazardous to human include lead, cadmium, mercury, arsenic, copper, zinc, and chromium. Arsenic and cadmium can cause cancer. Mercury can cause mutations and genetic damage, while copper, lead, and chromium can cause brain and bone damage. Heavy metals are often derived from industries such as electroplating and battery factories, metal finishing, and chemical manufacturing (Apiratikul et al. 2004).

The removal and recovery of heavy metal ions from wastewater involve many techniques such as ion exchange, evaporation, precipitation, membrane separation, etc. However, these common techniques are too expensive to treat low levels of heavy metals in wastewater. In addition, they have some disadvantages such as requiring a large area of lands, a sludge dewatering facility, skillful operators, and multiple basin configurations (Zhou et al. 1999). For example, the most serious limitation of ion exchange is the cost of resin. The price of resin is ranging from US$30 to 60/kg.

Biosorption is a process which utilizes inexpensive dead biomass to sequester toxic heavy metals (Kratochvil and Volesky 1998). Biosorption is proven to be quite effective at removing metal ions from contaminated solutions in a low-cost and environment-friendly manner. Herrera et al. (2004) found that an approximate cost of biosorption 10 g of Ag(II) onto cellulose phosphate was about US$2. Additionally, low-cost biosorption process using algae as adsorbent has lately been introduced as an alternative (unit cost of virgin algae is approximately ranging from US$1 to 3/kg).

Various dead biomasses were used as biosorbent for different toxic materials. The use of dead cells in biosorption has many advantages; dead cells are not affected by toxic wastes and do not require a continuous supply of nutrients. They can be regenerated and reused from many cycles. Dead cells may be stored or used for extended periods at room temperature without putrefaction occurring. Moreover, dead cells have shown to accumulate pollutants to the same or greater extended than growing cell (Fu and Viraraghavan 2002).

Biosorbents are prepared from the naturally abundant and/or waste biomass which has the ability to sequester heavy metals; these biosorbents are: yeast (Padmavathy 2008), bacteria (Sulaymon et al. 2012), algae (Rathinam et al. 2010), and fungi (Holan and Volesky 1995).

Algal biomass has proven to be highly effective as well as reliable and predictable in the removal of heavy metals from aqueous solutions. The term algae refers to a large and diverse assemblage of organisms that contain chlorophyll and carry out oxygenic photosynthesis (Davis et al. 2003). There are seven divisions of algae; four of which contain algae as members. Divisions which include the larger visible algae are: Cyanophyta (blue–green algae), Chlorophyta (green algae), Rhodophyta (red algae), and Phaeophyta (brown algae). These divisions are subdivided into orders, which are subsequently divided into families and then into genus and species (Naja et al. 2010).

The metal ion-binding mechanism in biosorption may involve different processes such as complexation, coordination, electrostatic attraction, or microprecipitation; whereby ion exchange plays a major role in the binding of metal ions by algae biomass. Therefore, the use of ion exchange reaction model instead of Langmuir- or Freundlich-type sorption isotherm has been recommended for describing the process (Schiwer and Volesky 1996).

The aim of the present research is to investigate the experimental and theoretical removal of lead, cadmium, copper, and arsenic ions as single, binary, ternary, and quaternary from simulated wastewater using algae as a biosorbents. Batch experiments were carried out for kinetic studies on the removal of those ions from aqueous solution. The influence of various important parameters such as pH, contact time, agitation speed, adsorbent dose, and initial concentration is investigated.

## Theoretical background

Several biosorption studies were done to develop a mathematical equilibrium sorption models and to verify their suitability for describing biosorption of heavy metals by algae biomass. These studies concluded that the biosorption mechanisms involving algae are an ion exchange reaction type between cations (light metals: Ca^2+^, Mg^2+^, Na^+^, and K^+^) already bound to the algae and other metals present in the aqueous solution (Naja and Volesky 2006; Diniz and Volesky 2005; Gin et al. 2002). In this case, light metal ions are initially attached to the sorbent binding sites (the functional groups) and the heavy metal ions are present in the solution. It has been demonstrated that the binding of metals by algal biomass from aqueous solutions can be described by ion exchange reaction (Kratochvil 1997):

$$ {{\mathrm{M}}^{2 + }} + \left( {{\mathrm{L}} - {\mathrm{biomass}}} \right) \leftrightarrow \left( {{\mathrm{M}} - {\mathrm{biomass}}} \right) + {{\mathrm{L}}^{2 + }} $$

where M^2+^ and L^2+^ represent the divalent metal cations sorbed and released from the biomass.

The total normality, which represents the sum of the equivalent concentrations of all competing cations that can be exchanged during the reaction, remains the same when equilibrium is achieved; hence, the total normality is expressed by:

1 $$ {c^0} = {c_{\mathrm{M}}} + {c_{\mathrm{L}}}. $$

In addition, if ions are exchanged during the process, the exchangeable binding sites are always occupied by the competing ions; thus, the total number of exchangeable binding sites is the sum of the concentrations in the solid phase of the elements involved and can be represented as follows:

2 $$ Q = {q_{\mathrm{M}}} + {q_{\mathrm{L}}}. $$

The equivalent fraction of one component in the liquid phase (*x*_M_, *x*_L_) is the ratio between its own concentration and the total normality of the solution, whereas the equivalent fraction in the solid phase (*y*_M_, *y*_L_) is its active concentration in the solid divided by the number of exchangeable binding sites:

3 $$ {x_{\mathrm{M}}} = \frac{{{c_{\mathrm{M}}}}}{{{c^{0\prime }}}}\quad {x_{\mathrm{L}}} = \frac{{{c_{\mathrm{L}}}}}{{{c^0}}} $$

4 $$ {y_{\mathrm{M}}} = \frac{{{q_{\mathrm{M}}}}}{Q},{y_{\mathrm{L}}} = \frac{{{q_{\mathrm{L}}}}}{Q} $$

For a single system, the affinity constant (*K*_M, L_) represents, in this case, the relative selectivity of the metal to the light ions and it is defined by (Diniz et al. 2008):

5 $$ {K_{{\mathrm{M}},{\mathrm{L}}}} = \frac{{{y_{\mathrm{M}}} \cdot {x_{\mathrm{L}}}}}{{{x_{\mathrm{M}}} \cdot {y_{\mathrm{L}}}}} $$

where the subscripts M and L are referring to the heavy metal and light metal in the solution.

Rearranging Eq. (5) by eliminating the light metal equivalent fraction, the model equation for the equilibrium uptake of a heavy metal ion present in a binary system with light metals can be written in the form of the following equation:

6 $$ {y_{\mathrm{M}}} = \frac{{{K_{{\mathrm{M}},{\mathrm{L}}}} \cdot {x_{\mathrm{M}}}}}{{1 + \left( {{K_{{\mathrm{M}},{\mathrm{L}}}} - 1} \right) \cdot {x_{\mathrm{M}}}}}. $$

Equation (6) represents an ion exchange isotherm for a single sorption system; the biosorption equilibrium data were set for heavy metal/light metals, where the first element indicates the sorbing metal, and the light metals specify the total amount of light metals released due to metal biosorption. The fraction of *y*_M_ and *x*_M_ is calculated from the experimental data and the affinity constant *K*_M,L_ is calculated from Eq.(6) by using the STATISTICA computer program. The higher *K*_M,L_ value means higher affinity of ions towards the adsorbent.

For a binary, ternary, and quaternary systems, Eqs. (7), (8), and (9) were used for each system, respectively.

7 $$ {y_{{{\mathrm{M}}_1}}} = \frac{{{K_{{{\mathrm{M}}_1},{\mathrm{L}}}} \cdot {x_{{{\mathrm{M}}_1}}}}}{{1 + \left( {{K_{{{\mathrm{M}}_1},{\mathrm{L}}}} - 1} \right) \cdot {x_{{{\mathrm{M}}_1}}} + \left( {{K_{{{\mathrm{M}}_2},{\mathrm{L}}}} - 1} \right) \cdot {x_{{{\mathrm{M}}_2}}}}} $$

8 $$ {y_{{{\mathrm{M}}_1}}} = \frac{{{K_{{{\mathrm{M}}_{1}},{\mathrm{L}}}} \cdot {x_{{{\mathrm{M}}_1}}}}}{{1 + \left( {{K_{{{\mathrm{M}}_{1}},{\mathrm{L}}}} - 1} \right) \cdot {x_{{{\mathrm{M}}_{1}}}} + \left( {{K_{{{\mathrm{M}}_{2}},{\mathrm{L}}}} - 1} \right) \cdot {x_{{{\mathrm{M}}_{2}}}}\left( {{K_{{{\mathrm{M}}_{3}},{\mathrm{L}}}} - 1} \right) \cdot {x_{{{\mathrm{M}}_{3}}}}}} $$

9 $$ {y_{{{\mathrm{M}}_1}}} = \frac{{{K_{{{\mathrm{M}}_{1}},{\mathrm{L}}}} \cdot {x_{{{\mathrm{M}}_{1}}}}}}{{1 + \left( {{K_{{{\mathrm{M}}_{1}},{\mathrm{L}}}} - 1} \right) \cdot {x_{{{\mathrm{M}}_{1}}}} + \left( {{K_{{{\mathrm{M}}_{2}},{\mathrm{L}}}} - 1} \right) \cdot {x_{{{\mathrm{M}}_{2}}}}\left( {{K_{{{\mathrm{M}}_{3}},{\mathrm{L}}}} - 1} \right) \cdot {x_{{{\mathrm{M}}_{3}}}} + \left( {{K_{{{\mathrm{M}}_{4}},{\mathrm{L}}}} - 1} \right) \cdot {x_{{{\mathrm{M}}_{4}}}}}} $$

## Experiments and materials

### Biomass and heavy metals

Various green (Chlorophyta) and blue–green (Cyanophyta) algae were used as biosorbent for the removal of Pb^2+^, Cd^2+^, Cu^2+^, and As^3+^ ions. The algae were collected in April and September 2011 from the Tigris River, Iraq. They were washed several times with tap water and then with deionized water to remove impurities and unwanted materials. The algae were analyzed by using microscope and their division, genus, and species were Cyanophyta (*Oscillatoria princeps* 92 %, *Oscillatoria subbrevis* 2 %, and *Oscillatoria formosa* 1 %) and Chlorophyta (*Spirogyra aequinoctialis* 3 %, *Mougeta* sp. 1 %, and others 1 %).

The algae biomass was sun-dried and then dried in oven at 50 °C for 24 h. The dried algae biomass was shredded, ground in a mortar, and sieved. An average size of 500–600 μm was used for biosorption experiments. Pb^2+^, Cd^2+^, Cu^2+^, and As^3+^ ion solutions were prepared by dissolving Pb(NO_3_)_2_·2H_2_O, Cd(NO_3_)_2_, Cu(NO_3_)_2_·3H_2_O, and As_2_O_3_ in distilled water. These solutions were kept at 25 °C. Concentrations of 50 ppm from these salts were used as adsorbate for different weight of algae biomass. The pH of solutions was adjusted to the required value using 0.1 M HNO_3_ and 0.1 M NaOH solutions. A pH meter type WTW/inoLab series was used. All chemicals used in this work were analytical reagent grade and were used without further purification. The solubility of Pb(NO_3_)_2_·2H_2_O, Cd(NO_3_)_2_, Cu(NO_3_)_2_·3H_2_O, and As_2_O_3_ in water is 54.3, 136, 125, and 1.8 g/100 g H_2_O, respectively.

### Biosorbent batch experiments

Batch experiments were carried out in 250 ml flasks containing 0.05, 0.1, 0.3, 0.5, 0.8, 1, 2, and 3 g of algae biomass and 100 ml of each solution. These experiments were performed at the same initial concentration for each element (50 ppm) for single and polymetallic systems. Different initial pH (2, 3, 4, 5, and 6) was used for each solution. The pH values were chosen below the precipitation value of each metal. The flasks were placed in a shaker (Edmund Buhler, 7400 Tubingen Shaker-SM 25) with constant shaking at 200 rpm for 4 h at 25 °C. Biosorbent was separated using centrifuge and filtration. The residual concentration of lead, cadmium, copper, arsenic, and light metals in solution was determined using atomic absorption spectrophotometer (type: Shimadzu, AAS 7200, Japan). The uptake (*q*_e_) is calculated for each metal using the following equation (Volesky 2003):

10 $$ {q_{\mathrm{e}}} = \frac{{\left( {{C_{\mathrm{i}}} - {C_{\mathrm{e}}}} \right)V}}{w}. $$

The experimental works were carried out to plot the isotherm curves by changing the weights of adsorbent and keeping constant adsorbate concentration at 50 ppm for single and polymetallic systems.

### Adsorption kinetic

A number of kinetic models have been used to describe the adsorption rate in batch operation. Pseudo-first-order kinetics model is (Sivakumar and Palanisamy 2010):

11 $$ \frac{{{\mathrm{d}}{q_{\mathrm{t}}}}}{\mathrm{dt}} = {k_1}\left( {{q_{\mathrm{e}}} - {q_{\mathrm{t}}}} \right) $$

where *q*_e_ and *q*_t_ are the amount of solute (in milligrams per gram) adsorbed onto the adsorbent at equilibrium and at time *t*, respectively, and *k*_1_ is the rate constant (in minute). Integrating and applying the boundary conditions, *q*_t_ = 0 at *t* = 0 and *q*_t_ = *q*_e_ at *t* = *t*, Eq. (11) takes the following form:

12 $$ \ln \left( {{q_{\mathrm{e}}} - {q_{\mathrm{t}}}} \right) = \ln \;{q_{\mathrm{e}}} - {k_1} \cdot t. $$

While the linearized form of the pseudo-second-order equation is (Fu and Viraraghavan 2002):

13 $$ \frac{t}{{{q_{\mathrm{t}}}}} = \frac{1}{{{k_2} \cdot {q_{\mathrm{e}}}}} + \frac{t}{{{q_{\mathrm{e}}}}} $$

where *k*_2_ is the rate constant of pseudo-second-order biosorption (in milligrams per gram), *q*_e_ is the amount of metal adsorbed at equilibrium (in milligrams per gram), and *q*_t_ is the amount of metal adsorbed at time *t* (in milligrams per gram).

Adsorption kinetic experiments were carried out by agitating 1 l of lead, cadmium, copper, and arsenic solutions of 50 ppm initial concentration. The dosage of algae to reach equilibrium concentration (*C*/*C*_i_) equals to 0.1 was calculated by using Eq. (10). Beaker of 2 l is filled with 1 l of solution and agitation is started before adding the optimum weight of algae, and then, samples were taken for each 1, 2, 3, 5, 10, 15, 20, 25, 30, 40, 50, 60, 80, 100, 120, 150, 180, 210, and 240 min. The optimum pH of removal of each solution obtained from equilibrium isotherm experiments was fixed for each solution before agitation process was started.

## Results and discussion

### Fourier transformation infrared spectroscopy analysis

Many authors have used Fourier transformation infrared (FTIR) spectroscopy to detect vibration frequency changes in algae. FTIR offers excellent information on the nature of the bands present on the surface of the algae and also presents three main advantages as an analytical technique: it is fast, nondestructive, and requires only small sample quantities (Pereira et al. 2003).

The characteristics of absorption bands of hydroxyl and amine groups were identified at 3,414 cm^−1^, alkyl chains at 2,966 and 2,943 cm^−1^, C=0 of the carboxylic groups or ester groups at 1,797, C=O of amide groups at 1,647 cm^−1^, COO^−^ of the carboxylate groups appeared at 1,427 cm^−1^, S=O of the sulfonate groups and COO^−^ groups of the fatty acids at 1,300 cm^−1^, and the wave number at 1,033 cm^−1^ were attributed to the P–O–C links of the organic phosphate groups (Naja et al. 2005, 2010). Some bands in the fingerprint regions could be attributed to the phosphate groups (Diniz et al. 2008).

**Fig. 1:**
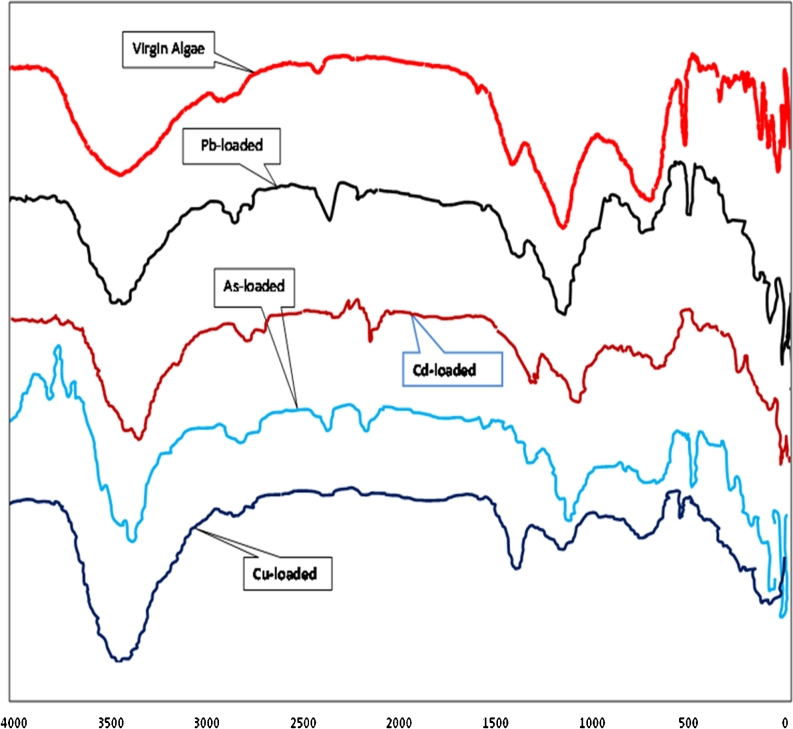
FTIR spectra obtained for virgin algae and Pb^2+^-, Cd^2+^-, Cu^2+^-, and As^3+^-loaded algae

Four flasks of 250 ml were filled with 100 ml of each metal solution (50 ppm) and 1 g of dried algae. The flasks were then placed on a shaker and agitated continuously for 4 h at 200 rpm. Infrared spectra of dried algal biomass samples before and after Pb^2+^, Cd^2+^, Cu^2+^, and As^3+^ biosorption were examined using (Shimadzu FTIR, 800 Series Spectrophotometer). Figure 1 shows the results of FTIR analyses of virgin algae (before biosorption) and Pb^2+^-, Cd^2+^-, Cu^2+^-, and As^3+^-loaded algae. From this figure, the main functional groups that are responsible for the metal uptake onto algal biomass were carboxyl, sulfonates, and hydroxyl, mainly those from polysaccharidic material which constitutes most of the algal biomass surface wall (Davis et al. 2003; Naja et al. 2010; Schiwer and Volesky 1996)

The percent transmittance of peaks bands before and after biosorption was observed at different values mentioned in Table 1. In this table, it can be observed that the percent transmittance values were shifted due to biosorption, and Pb^2+^ has greatest changes in the percent transmittance values of bands than As^3+^ and Cu^2+^, while Cd^2+^ was the lowest one.

**Table 1:** Values of observed peak bands for virgin algae and Pb^2+^-, Cd^2+^-, Cu^2+^-, and As^3+^-loaded algae

Band	% transmittance				
	Virgin algae	Pb^2+^ loaded	Cd^2+^ loaded	Cu^2+^ loaded	As^3+^ loaded
3,414	52	77	54	42	73
2,966	78	78	68	86	85
2,515	88	91	70	92	85
1,797	80	89	69	91	85
1,647	55	83	62	72	83
1,427	32	76	59	78	72
1,238	62	87	66	85	82
1,033	42	83	63	80	81
875	61	85	66	85	83
821	92	94	70	90	88
713	76	83	66	84	83
532	64	72	62	60	76
459	52	66	52	60	65

**Fig. 2:**
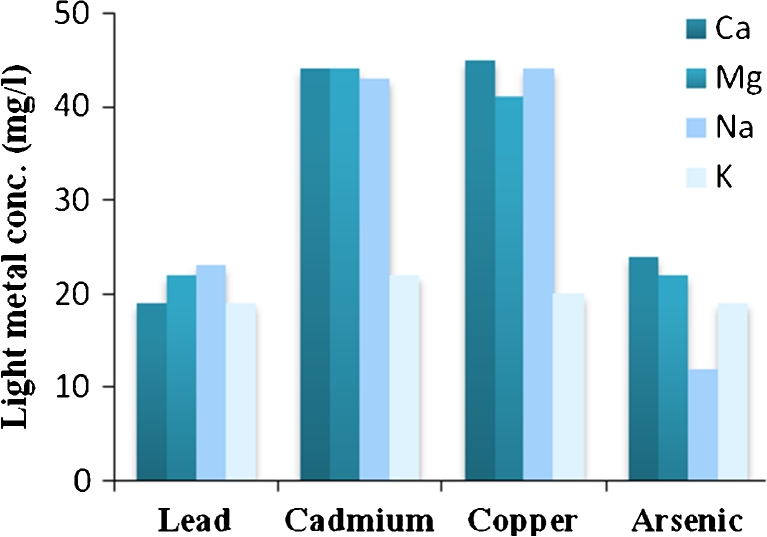
Amounts of light metals released due to heavy metal biosorption, *C*_i_ = 50 ppm, contact time 4 h at 200 rpm

Ion exchange mechanism was previously demonstrated as the main mechanism involved in heavy metal uptake by algal biosorbent materials. In order to confirm this phenomenon in the present work and to identify the light metals that bounded to the surface functional groups of the algal biomass, the concentration of light metals that released due to the heavy metal biosorption was found. Figure 2 shows the amount of light metals released due to biosorption of 50 ppm of four heavy metals onto 1 g of algal biomass at pH = 4.

### Effect of pH

In the biosorption process, the pH affects two aspects: metal ion solubility and biosorbent total charge, since protons can be adsorbed or released (Romera et al. 2007). This behavior will depend on the functional groups present on the alga cell wall. Therefore, the pH value of the medium affects the system's equilibrium state, according to the following equations.

14 $$ {\mathrm{B}} - {\mathrm{H}} \leftrightarrow {{\mathrm{B}}^- } + {{\mathrm{H}}^+ } $$

where *K*_a_ is given by

15 $$ {K_{\mathrm{a}}} = \frac{{\left[ {{{\mathrm{B}}^- }} \right]\left[ {{{\mathrm{H}}^+ }} \right]}}{{\left[ {{\mathrm{B}} - {\mathrm{H}}} \right]}} $$

16 $$ {\mathrm{p}}{K_{\mathrm{a}}} - {\mathrm{pH}} = \log \frac{{\left[ {{\mathrm{B}} - {\mathrm{H}}} \right]}}{{\left[ {{{\mathrm{B}}^- }} \right]}} $$

For pH values lower than p*K*_a_, equilibrium (14) shifts to the left, consuming protons and increasing pH until its value equals the p*K*_a_, the opposite will happen.

**Fig. 3:**
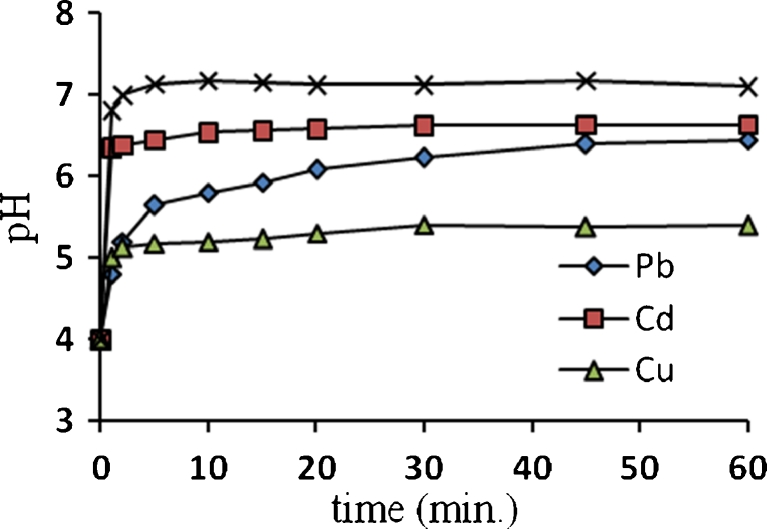
pH evolution as a function of time, and initial pH is 4 for all solutions

**Fig. 4:**
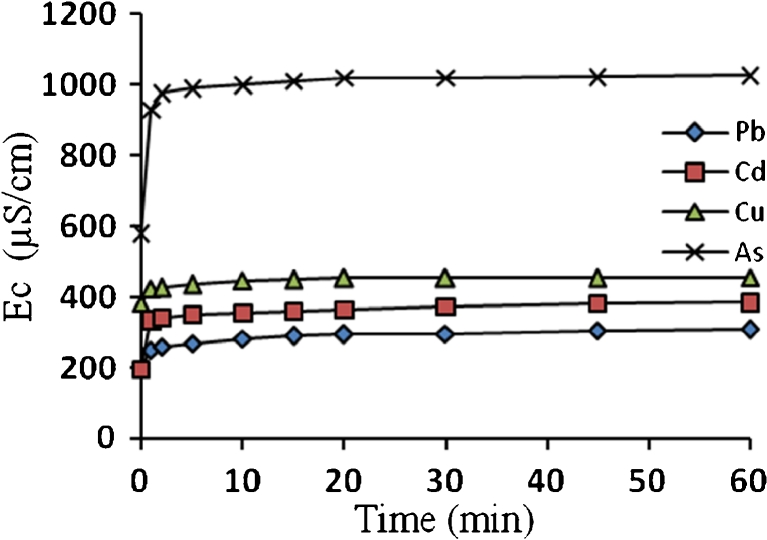
Electrical conductivity evolution as a function of time

Untreated algae biomass generally contains alkali and alkaline earth metals such as K^+^, Na^+^, Ca^2+^, and Mg^2+^ which are originally present in sea- and freshwater. These ions are bound to the surface acidic functional groups. It has been reported that when algae biomass reacts with heavy metal-bearing solution, pH increases and releases light metal ions. This also was explained in terms of ion exchange, whereby the observed released light metals balanced the uptake of heavy metals (Schiwer and Volesky 1996; Kratochvil 1997). The release of these light metals will cause the increasing of pH and electrical conductivity of the solution. Figures 3 and 4 show the increase in the pH and electrical conductivity, respectively (1 h biosorption process of Pb^2+^, Cd^2+^, Cu^2+^, and As^3+^ ions, with an initial pH of 4 for each solution).

**Fig. 5:**
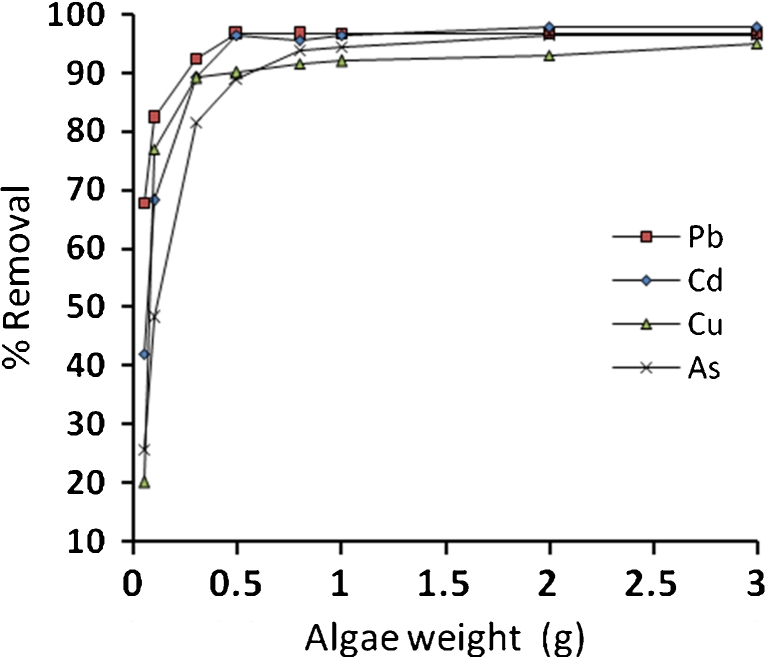
Biosorption efficiency at optimum pH value for Pb^2+^, Cd^2+^, Cu^2+^, and As^3+^

In order to examine the effects of pH on the Pb^2+^, Cd^2+^, Cu^2+^, and As^3+^ ion removal efficiency, several experiments were carried out at various initial pH values (from 2 to 6) with different amounts of algae (0.05–3 g) in a series of flasks containing 100 ml of heavy metal solutions (50 mg/l) which were agitated at 200 rpm for 4 h. Then, the concentration of heavy metals after adsorption was determined. The experimental data showed that the optimum pH for removal was 3, 5, 4, and 5 for Pb^2+^, Cd^2+^, Cu^2+^, and As^3+^ ions, respectively. Therefore, the best removal occurs at a pH that ranged from 3 to 5 for all metals; this is in good agreement with Davis et al. (2003), Romera et al. (2007), and Jinbai (1999). At pH below 2.5, the positive charge (H^+^) density on the sites of biomass surface minimizes metal sorption, and above 6, metal precipitations is favored. Apiratikul et al. (2004) found that for Pb^2+^, Cd^2+^, and Cu^2+^, the pH values of precipitation are >7, >6.3, and >6, respectively. Figure 5 shows the biosorption efficiency at optimum pH value for Pb^2+^, Cd^2+^, Cu^2+^, and As^3+^, respectively.

### Effect of initial concentration

**Fig. 6:**
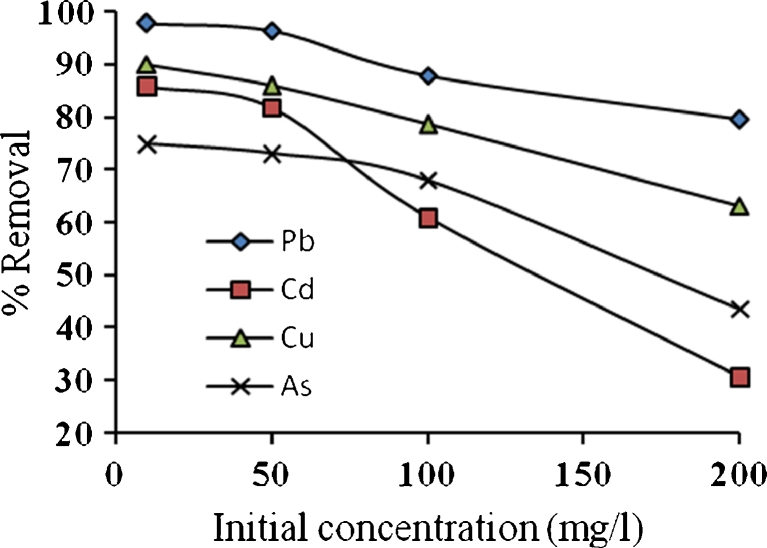
Variation of percentage removal with concentration, pH = 4, *t* = 20 °C

Different concentrations (10, 50, 100, and 200 mg/l) were selected to study the variation of removal efficiency with different initial concentration at the same weight of algae biomass (1 g) at 20 °C. The pH of solutions was fixed at 4 and the agitation speed of shaker was 200 rpm for 4 h. The results are plotted in Fig. 6; it can be seen that the percentage removal does not alter greatly if the concentration increases from 10 to 50 mg/l. This behavior is due to 1 g of algae that may contain enough exchangeable sites for this concentration range, but when the concentrations increase to 100 and 200 mg/l, the exchangeable sites in 1 g will not be enough to cover these concentrations so that the depletion in percentage removal will be obvious.

### Effect of temperature

**Fig. 7:**
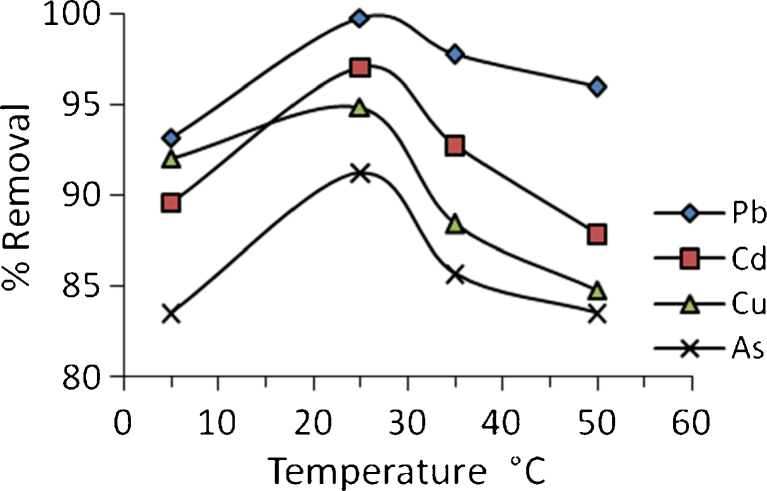
Variation of percentage removal with temperature, pH = 4, *C*_i_ = 50 mg/l for each metal

The effect of temperature on the equilibrium sorption capacity for Pb^2+^, Cd^2+^, Cu^2+^, and As^3+^ ions has been investigated at temperature 15–35 °C with initial heavy metal concentration of 50 mg/l and pH 4. Figure 7 shows the variation of percentage removal with temperature of solution. It can be concluded that a maximum percentage removal of four metals has been obtained at 25 °C. This suggests that biosorption between algal biomass and metals could involve a combination of chemical interaction and physical adsorption. With an increase in temperature above 5 to 25 °C, pores in the algae enlarge resulting in an increase of the surface area available for the sorption, diffusion, and penetration of metal ions within the pores of algae causing an increase in sorption (Saleem et al. 2007). Also, increasing temperature is known to increase the diffusion rate of adsorbate molecules within pores as a result of decreasing solution viscosity and will also modify the equilibrium capacity of the adsorbent for a particular adsorbate.

Further increase in temperature (above 25 °C) leads to a decrease in the percentage removal. This decrease in biosorption efficiency may be attributed to many reasons: increasing in the relative escaping tendency of the heavy metals from the solid phase to the bulk phase, deactivating the biosorbent surface, or destructing some active sites on the biosorbent surface due to bond ruptures (Meena et al. 2005) or due to the weakness of biosorption forces between the active sites of the sorbents and the sorbate species and also between the adjacent molecules of the sorbed phase (Ahmet and Mustafa 2008).

### Biosorption isotherms

Differentiating between single and competitive biosorption systems must be considered. In competitive systems, binding of different metal ions on biomaterials having different functional groups depends on ionic properties such as electronegativity, ionic radius, potential, and redox potential of these metals (Naja et al. 2010). Sulaymon et al. (2011) showed that large ionic radius (molecular cross-sectional area) resulted in greater adsorption efficiency. Allen and Brown (1995) proposed that more electronegative metal ions will be more strongly attracted to the surface. Table 2 shows the ionic properties of Pb^2+^, Cd^2+^, Cu^2+^, and As^3+^.

**Table 2:** Ionic properties of Pb^2+^, Cd^2+^, Cu^2+^, and As^3+^

Metal	Atomic radius (A°)	Electronegativity (Pauling's)	Ionization energy (kcal/g/mol)
Pb^2+^	1.75	1.8	171
Cd^2+^	1.54	1.7	207
Cu^2+^	1.28	1.9	178
As^3+^	1.39	2	231

Ion exchange was previously demonstrated as the main mechanism involved in heavy metal uptake by algal biosorbent materials. Hence, the isotherm data were fitted with ion exchange models Eqs. (6–9).

**Fig. 8:**
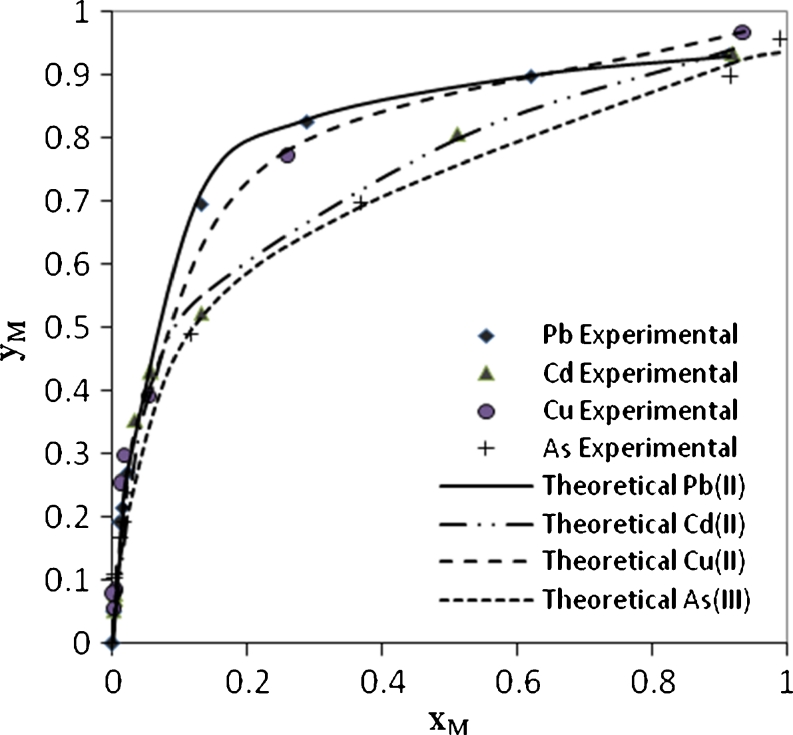
Ion exchange equilibrium isotherm for Pb^2+^, Cd^2+^, Cu^2+^ and As^3+^ in a single system

Figure 8 shows the experimental and theoretical (ion exchange) data for Pb^2+^, Cd^2+^, Cu^2+^, and As^3+^, respectively, in the single system. This figure shows a well fitting between the experimental and theoretical data due to high *R* ^2^ values, the calculated equilibrium constants (affinity constant) *K* for each metal obtained by nonlinear regression of experimental data with an ion exchange model using STATISTICA software ver. 6. All affinity constant values with correlation coefficient for single, binary, ternary, and quaternary systems are summarized in Table 3.

**Table 3:** Values of affinity constant (*K*) and *R* ^2^ for each metal system

Metal	System	*K*	*R* ^2^
Pb	Single (Pb)	16.55	0.995
	Binary (Pb+Cd)	8.765	0.988
	Binary (Pb+Cu)	11.64	0.936
	Binary (Pb+As)	7.77	0.994
	Ternary (Pb+Cd+Cu)	3.11	0.994
	Ternary (Pb+Cd+As)	3.89	0.978
	Ternary (Pb+Cu+As)	2.68	0.997
	Quaternary (Pb+Cd+Cu+As)	2.56	0.989
Cd	Single (Cd)	10.52	0.985
	Binary (Cd+Pb)	7.37	0.985
	Binary (Cd+Cu)	4.332	0.984
	Binary (Cd+As)	9.47	0.994
	Ternary (Cd+Pb+Cu)	4.298	0.998
	Ternary (Cd+Pb+As)	4.68	0.992
	Ternary (Cd+Cu+As)	5.6	0.997
	Quaternary (Cd+Pb+Cu+As)	1.3	0.995
Cu	Single (Cu)	15.97	0.988
	Binary (Cu+Pb)	4.511	0.932
	Binary (Cu+Cd)	3.97	0.961
	Binary (Cu+As)	5.45	0.966
	Ternary (Cu+Pb+Cd)	3.68	0.981
	Ternary (Cu+Pb+As)	2.56	0.992
	Ternary (Cu+Cd+As)	2.98	0.991
	Quaternary (Cu+Pb+Cd+As)	3.2	0.994
As	Single (As)	7.45	0.984
	Binary (As+Pb)	6.074	0.991
	Binary (As+Cd)	5.94	0.869
	Binary (As+Cu)	7.98	0.984
	Ternary (As+Pb+Cd)	4.15	0.977
	Ternary (As+Pb+Cu)	4.53	0.996
	Ternary (As+Cd+Cu)	6.09	0.996
	Quaternary (As+Pb+Cd+Cu)	4.74	0.993

Binary, ternary, and quaternary mixtures of heavy metals are usually present in effluent from different industries. As shown previously in single isotherm experiments, the pH of mixtures in binary, ternary, and quaternary systems was fixed at 4 since this value was correspondent for all metal removal.

The values of affinity constants gave a good indicator to understand the biosorption capacity of metals. For single system, the greatest values of *K* were 16.55 for Pb^2+^ then 15.97, 10.52, and 7.45 for Cu^2+^, Cd^2+^, and As^3+^, respectively.

**Fig. 9:**
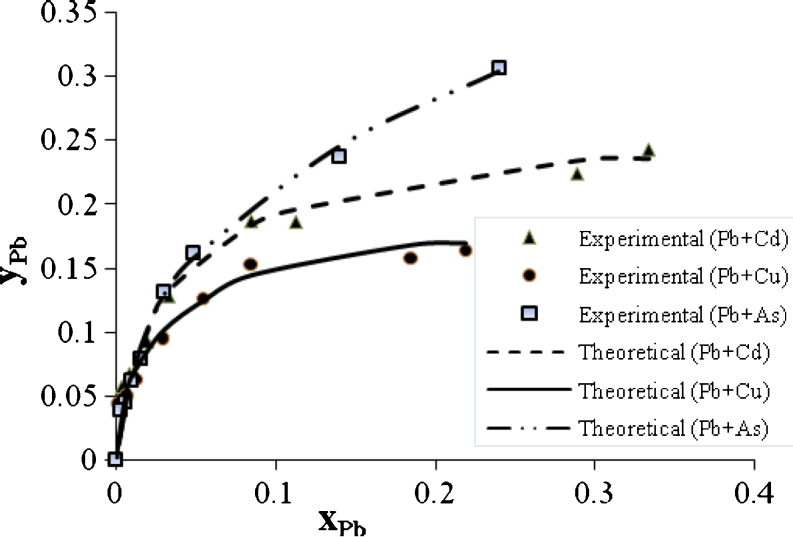
Ion exchange equilibrium isotherm for Pb^2+^ in a binary system

**Fig. 10:**
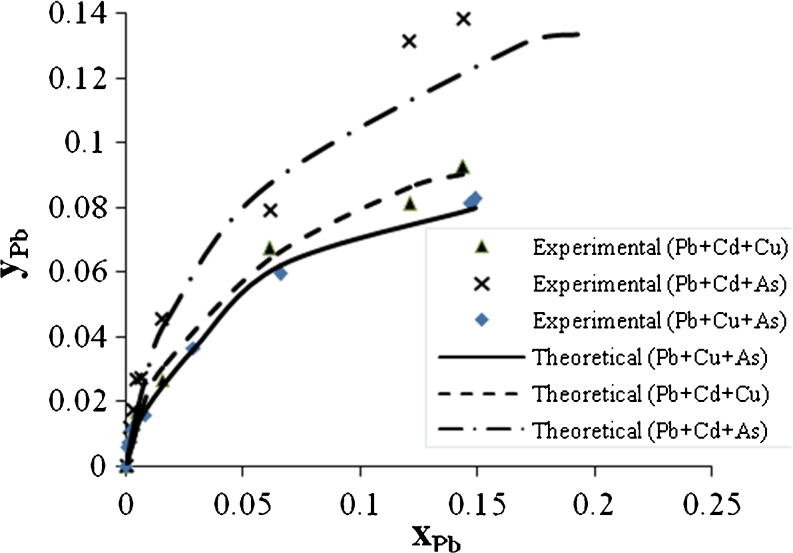
Ion exchange equilibrium isotherm for Pb^2+^ in a ternary system

**Fig. 11:**
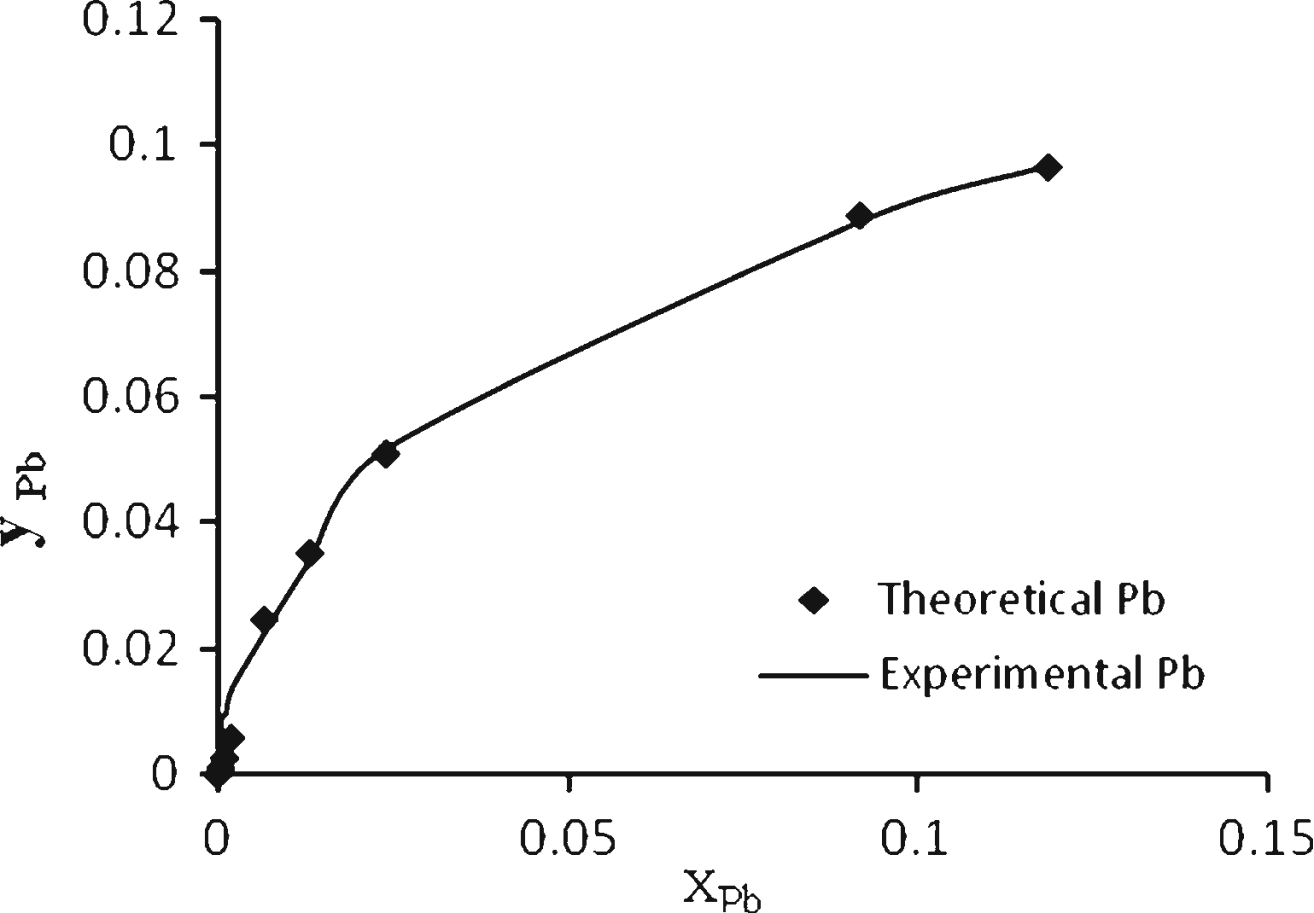
Ion exchange equilibrium isotherm for Pb^2+^ in quaternary system

Figures 9, 10, and 11 show an ion exchange equilibrium isotherm for Pb^2+^ in binary, ternary, and quaternary systems. It is clear that the As^3+^ ion has the greatest effect on the biosorption rate of Pb^2+^ in binary system than Cu^2+^ or Cd^2+^; this effect also remains in ternary and quaternary systems.

**Fig. 12:**
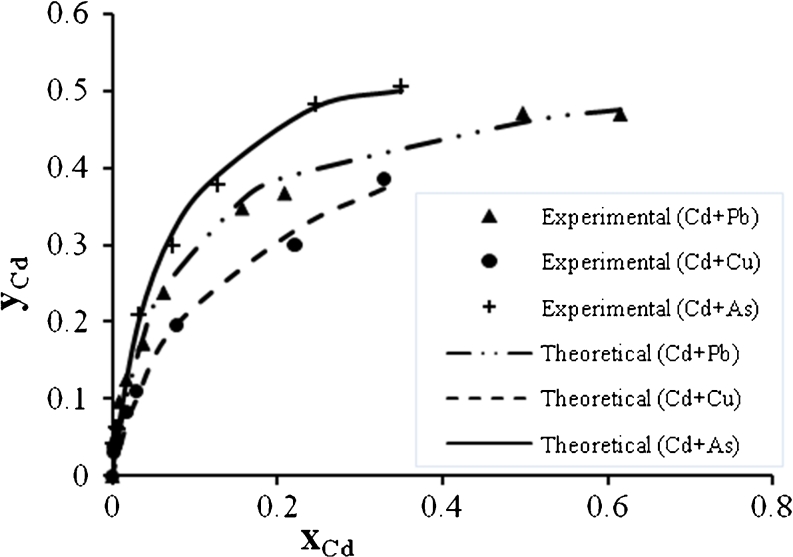
Ion exchange equilibrium isotherm for Cd^2+^ in a binary system

**Fig. 13:**
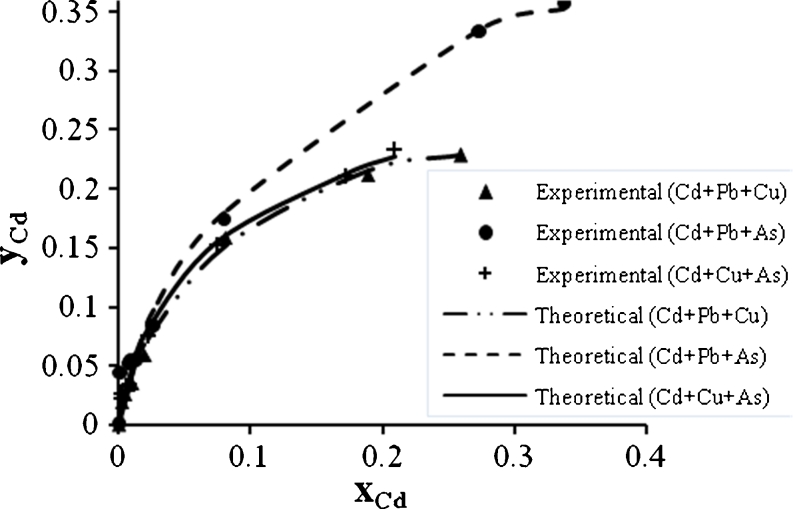
Ion exchange equilibrium isotherm for Cd^2+^ in a ternary system

**Fig. 14:**
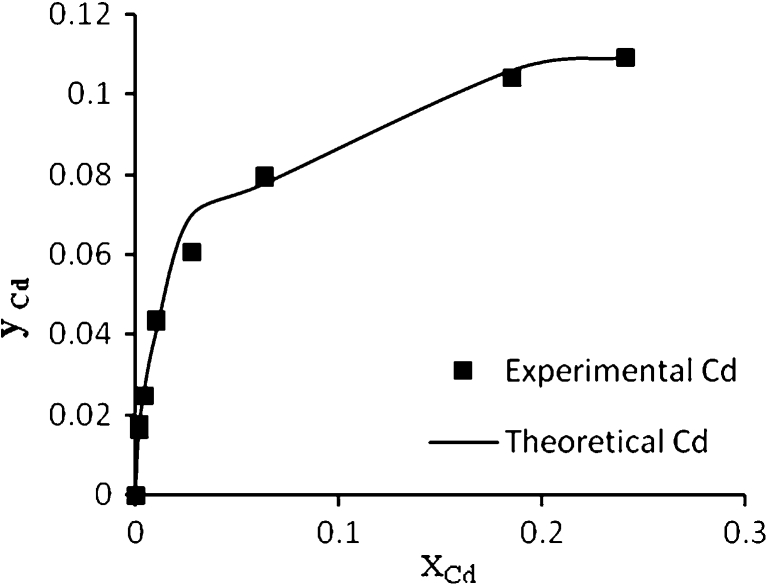
Ion exchange equilibrium isotherm for Cd^2+^ in quaternary system

Figures 12, 13, and 14 show the Cd^2+^ biosorption in binary, ternary, and quaternary systems. It is clear that the Cu^2+^ has the greatest effect on the Cd^2+^ biosorption rate in binary system than Pb^2+^ or As^3+^, but in ternary system, the Cd^2+^ mixture with Pb^2+^ and Cu^2+^ reduces the biosorption rate to the lowest value. The biosorption of Cd^2+^ was the lowest one when compared with the biosorption of other metals in quaternary system.

**Fig. 15:**
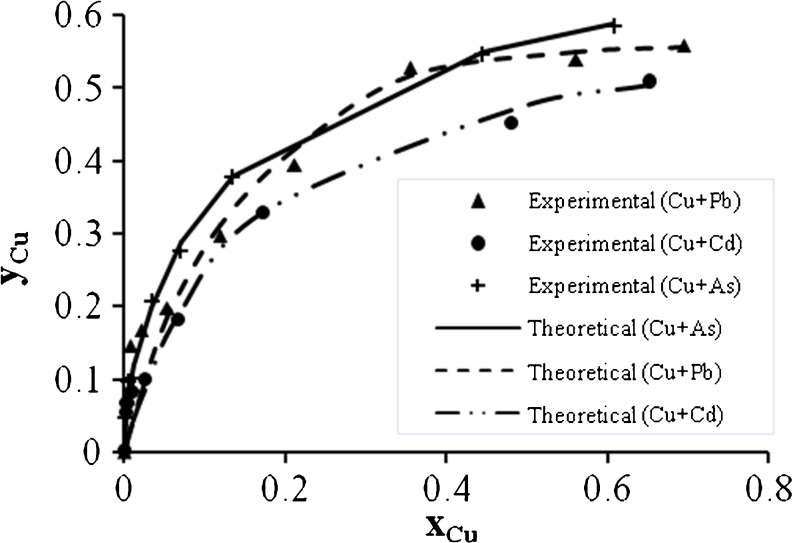
Ion exchange equilibrium isotherm for Cu^2+^ in a binary system

**Fig. 16:**
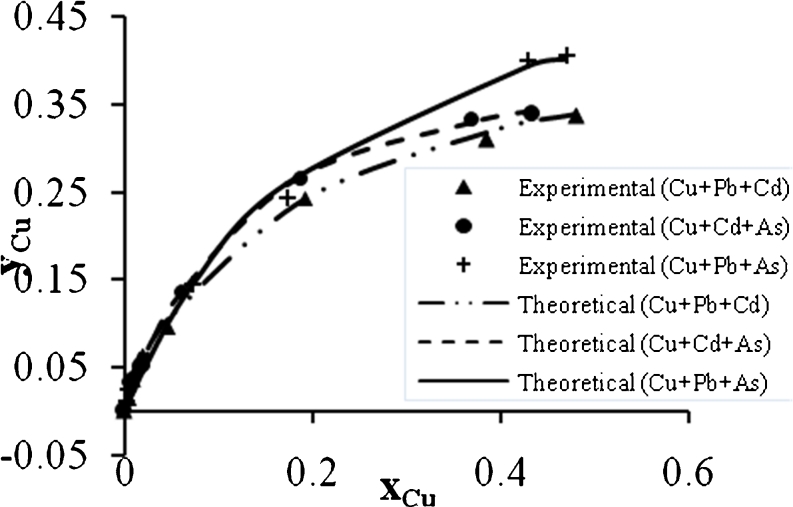
Ion exchange equilibrium isotherm for Cu^2+^ in a ternary system

**Fig. 17:**
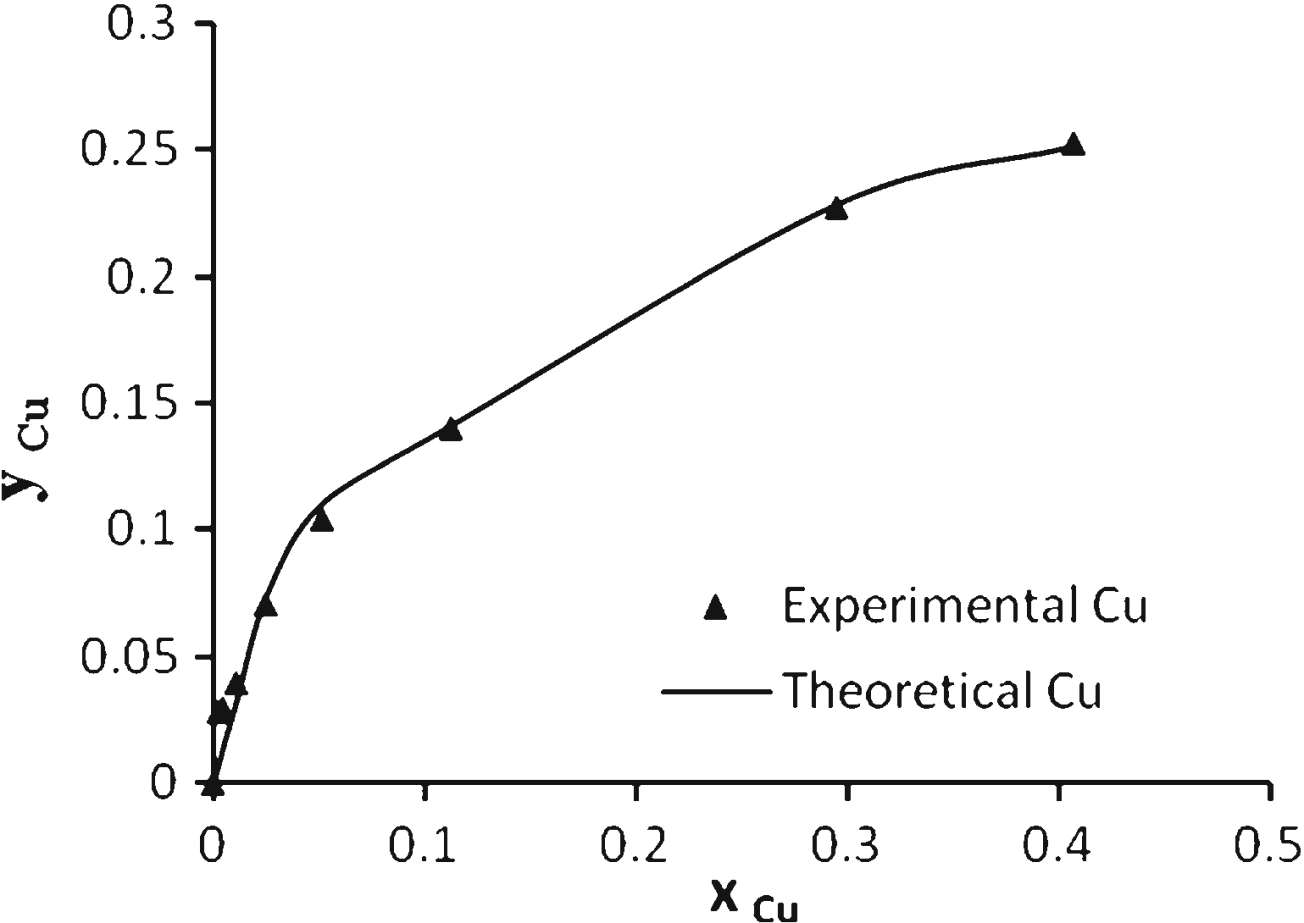
Ion exchange equilibrium isotherm for Cu^2+^ in quaternary system

Figures 15, 16, and 17 show the Cu^2+^ biosorption in binary, ternary, and quaternary systems. It can be seen that the mixing of Cu^2+^ with Cd^2+^ causes high reduction in the biosorption capacity. In addition, the ternary mixtures of Cu^2+^ with (Pb^2+^ + As^3+^) or (Cd^2+^ + As^3+^) reduce the biosorption capacity to a lower value compared with the other quaternary system.

**Fig. 18:**
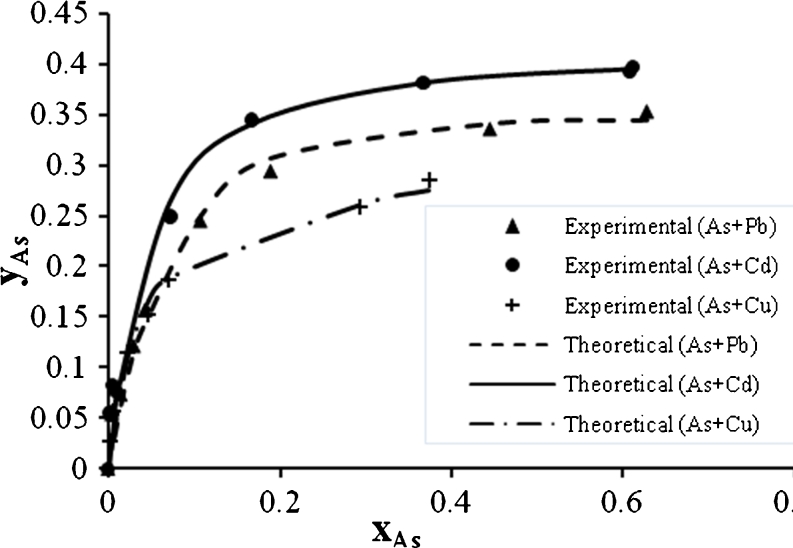
Ion exchange equilibrium isotherm for As^2+^ in a binary system

**Fig. 19:**
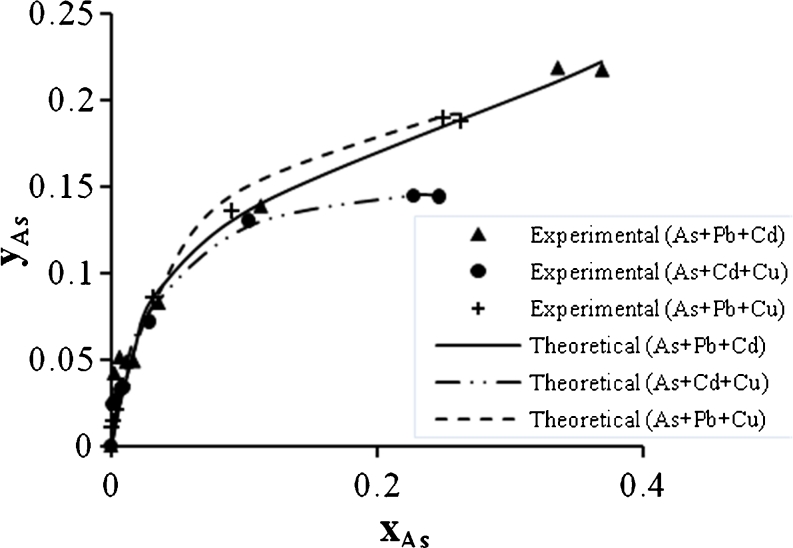
Ion exchange equilibrium isotherm for As^2+^ in a ternary system

**Fig. 20:**
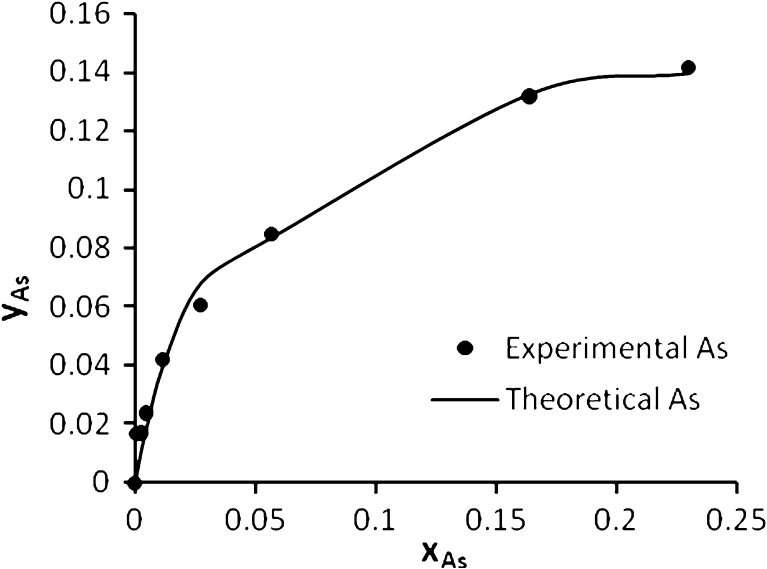
Ion exchange equilibrium isotherm for As^3+^ in quaternary system

Figures 18, 19, and 20 show the As^3+^ biosorption in binary, ternary, and quaternary systems. As^3+^ also has a high electronegativity so that it was expected that this metal will be affected in a high extent by mixing with Pb^2+^ as shown in (Fig. 5). But in the ternary system, this metal showed low biosorption when mixing with Cu^2+^ and Cd^2+^.

The decrease in affinity constant values in binary, ternary, and quaternary systems when compared with the single metal biosorption of four metals is due to the competition between metals for binding sites present in algal biomass wall. The biosorption capacity for each metal decreases when increasing the number of metals, so that at quaternary system, the lowest biosorption capacity was obtained.

### Optimum agitation speed

Vijayaraghavan and Yun (2008) indicated that with appropriate agitation, the mass transfer resistance can be minimized. Additionally, increasing the agitation rate, the diffusion rate of a solute from the bulk liquid to the liquid boundary layer surrounding particles becomes higher due to the enhanced turbulence and the decrease in the thickness of the liquid boundary layer. Under these conditions, the value of the external diffusion coefficient becomes larger.

**Fig. 21:**
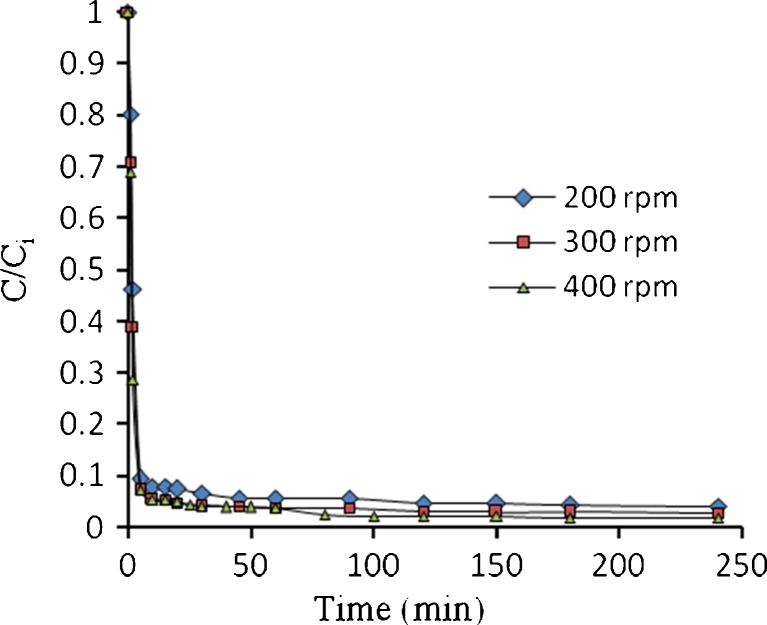
Concentration time decay curves for Pb^2+^ adsorption onto algae at different agitation speed

**Fig. 22:**
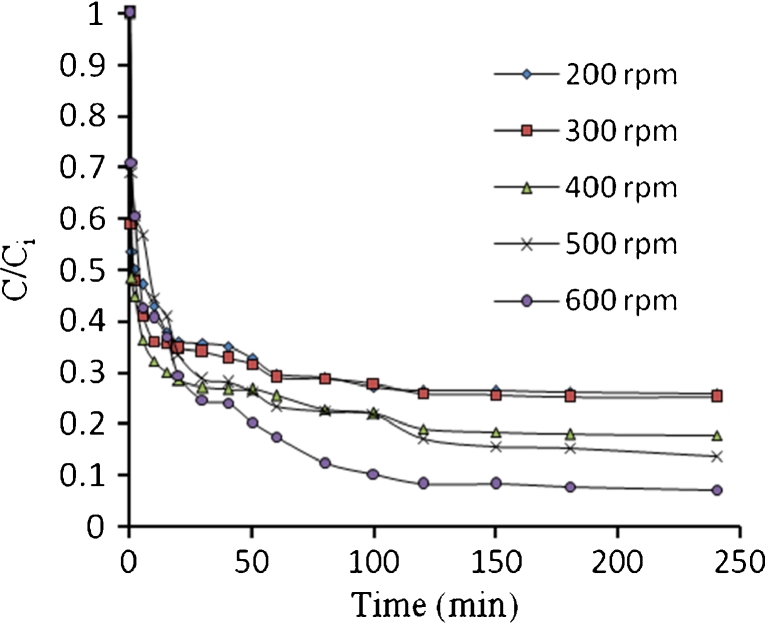
Concentration time decay curves for Cd^2+^ adsorption onto algae at different agitation speed

**Fig. 23:**
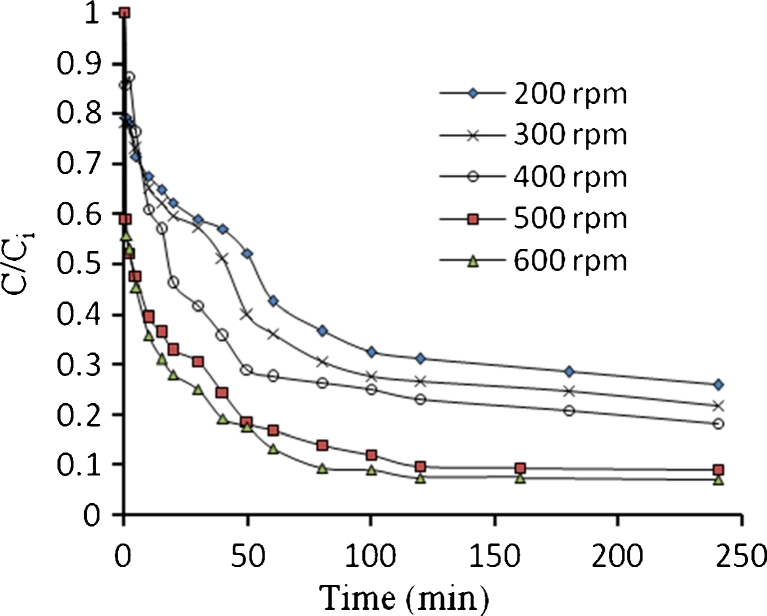
Concentration time decay curves for Cu^2+^ adsorption onto algae at different agitation speed

**Fig. 24:**
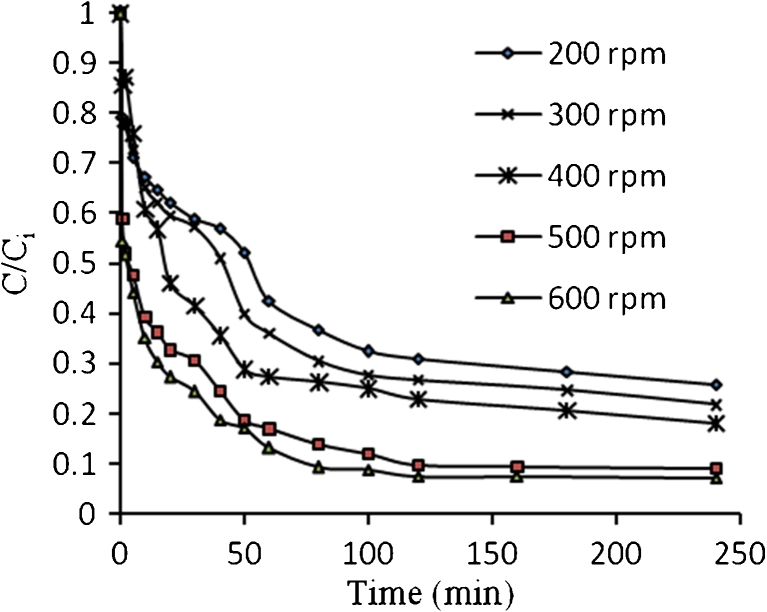
Concentration time decay curves for As^3+^ adsorption onto algae at different agitation speed

Figures 21, 22, 23, and 24 show the typical concentration decay curves of Pb^2+^, Cd^2+^, Cu^2+^, and As^3+^ solute in batch experiments carried out at different agitation speeds. The optimum agitation speed needed to achieve *C*/*C*_o_ = 0.1 was found to be 300, 600, 500, and 600 rpm for Pb^2+^, Cd^2+^, Cu^2+^, and As^3+^, respectively. Figure 15 shows a fast rate for lead removal in the first 10 min when compared with other metals due to high adsorption rate while slow rate was attributed to the interior penetration.

### Biosorption kinetics

The study of biosorption kinetics of heavy metal removal from wastewater is significant as it provides valuable insights into the reaction pathways and into the mechanism of sorption reactions. Monitoring a kinetic experiment helps to study how the biosorption system is affected by process variables and to understand the steps which limit biosorption. In addition, the biosorption kinetics describes the solute uptake rate which in turn controls the residence time of biosorbate uptake at the solid–solution interface. Therefore, it is important to predict the rate at which sorbate is removed from aqueous solutions in order to design appropriate sorption treatment processes.

**Fig. 25:**
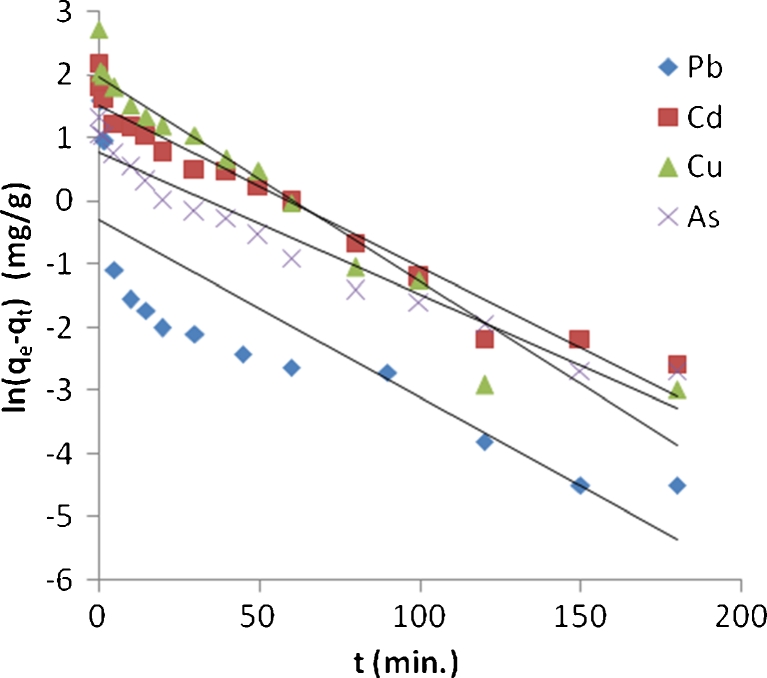
Pseudo-first-order kinetic model for biosorption of lead, cadmium, copper, and arsenic onto algal biomass

**Fig. 26:**
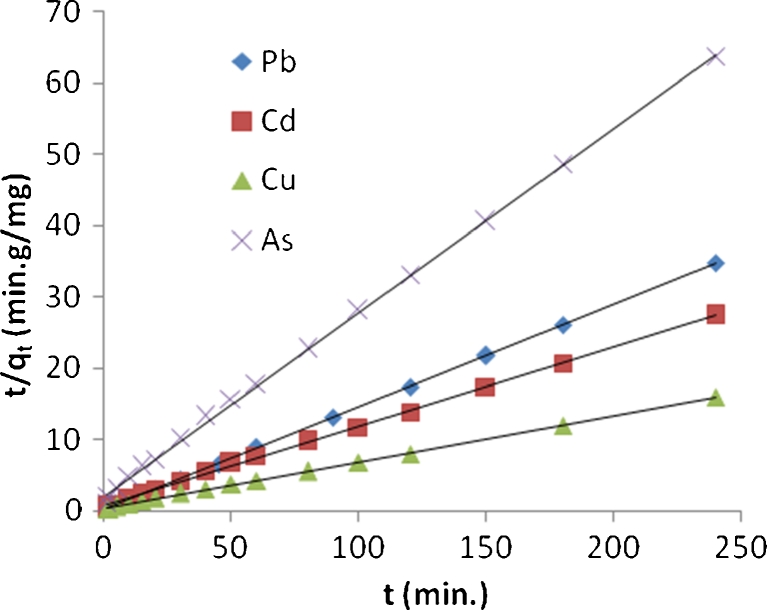
Pseudo-second-order kinetic model for biosorption of lead, cadmium, copper, and arsenic onto algal biomass

To analyze the mechanisms of biosorption process, the experimental data at optimum agitation speed were fitted to the pseudo-first- and pseudo-second-order kinetic models. The rate constant (*k*_1_) using pseudo-first-order model for Pb^2+^, Cd^2+^, Cu^2+^, and As^3+^ was obtained from the slope of the linear plots of ln(*q*_e_ − *q*_t_) against *t* for each solute using Eq. (12) (Fig. 25). The rate constant (*k*_2_) for these metals by using pseudo-second order was obtained from the slope and intercept of plots of *t*/*q*_t_ against *t* using Eq. (13) (Fig. 26).

The rate constants with the corresponding *R* ^2^ are presented in Table 4 for both mechanisms and for the four metal ions; according to these results, the experimental data for four metals followed pseudo-second order. It is clear that *R* ^2^ values for pseudo-second-order model are very high (0.998–0.999) when compared with pseudo-first-order model (0.687–0.957). These results suggest that this model successfully describes the kinetics of the biosorption of Pb^2+^, Cd^2+^, Cu^2+^, and As^3+^ ions onto algae biomass. This means that the biosorption of these metals occurs in a monolayer on the surface of adsorbent. This conclusion is in agreement with those obtained by others (Ahmet and Mustafa 2008; Mata et al. 2009).

**Table 4:** Calculated kinetic parameters for pseudo-first and pseudo-second order for Pb^2+^, Cd^2+^, Cu^2+^, and As^3+^ with correlation coefficients

Metal	Pseudo-first-order kinetic model		Pseudo-second-order kinetic model	
	*k*_1_ (1/min)	*R* ^2^	*k*_2_ (mg/g/min)	*R* ^2^
Pb^2+^	0.028	0.687	0.169	0.999
Cd^2+^	0.025	0.957	0.0177	0.998
Cu^2+^	0.032	0.939	0.727	0.999
As^3+^	0.022	0.946	0.0315	0.999

## Conclusions

The present study evaluated the competition removal of Pb^2+^, Cd^2+^, Cu^2+^, and As^3+^ using algae. The biosorption process depends significantly on the pH of the solution and is favored at around pH of 3–5.

The result showed that a well fitting exists between the ion exchange model and experimental data. The affinity constant sequence calculated for single system was *K*_Pb_ > *K*_Cu_ > *K*_Cd_ > *K*_As_; then, the affinity constant values reduce in binary and ternary systems, while the lowest value in the quaternary system is reached due to the competition among the four metals. The optimum agitation speed to reach 90 % removal efficiency was 300, 600, 500, and 600 rpm for Pb^2+^, Cd^2+^, Cu^2+^, and As^3+^, respectively. Kinetics investigation of the equilibrium data showed that the biosorption of Pb^2+^, Cd^2+^, Cu^2+^, and As^3+^ onto algae followed well the pseudo-second-order kinetic model.

## Nomenclature

*C*: Concentration of metal at any time (in milligrams per liter)
*C*_e_: Equilibrium concentration of metal ion (in milligrams per liter)
*C*_i_: Initial concentration of metal ion (in milligrams per liter)
*c*_L_: Light metal normality (in milliequivalents per liter)
*c*_M_: Heavy metal normality (in milliequivalents per liter)
*c*^0^: Total normality of solution (in milliequivalents per liter)
*K*: Affinity constant
*k*_1_: Adsorption rate constant of the pseudo-first-order equation (1/min)
*k*_2_: Adsorption rate constant of the pseudo-second-order equation (in kilograms per gram per minute)
*Q*: Total number of ion exchangeable binding sites (in milliequivalents per gram)
*q*_e_: Adsorption amount of metal ions (in milligrams per kilogram)
*q*_L_: Light metal concentration in the solid phase (in milliequivalents per gram)
*q*_M_: Heavy metal concentration in the solid phase (in milliequivalents per gram)
*q*_t_: Amount of metal ions adsorbed (in milligrams per kilogram)
*t*: Time (in minute)
*V*: Volume of solution (in liter)
*w*: Weight of adsorbent (in gram)
*x*_L_: Light metal equivalent fraction in the liquid
*x*_M_: Heavy metal equivalent fraction in the liquid
*y*_L_: Light metal equivalent fraction in the solid
*y*_M_: Heavy metal equivalent fraction in the solid
